# Comparison of the efficacy of repeated greater occipital nerve block and pulsed radiofrequency therapy in chronic migraine patients: a randomized controlled study

**DOI:** 10.22514/jofph.2024.031

**Published:** 2024-09-12

**Authors:** Esra Ertilav, Osman Nuri Aydın

**Affiliations:** ^1^Department of Neurology & Algology, Adnan Menderes University Medical Faculty, 09100 Aydin, Turkey; ^2^Department of Anesthesiology & Algology, Adnan Menderes University Medical Faculty, 09100 Aydin, Turkey

**Keywords:** Chronic migraine, Greater occipital nerve, Pulsed radiofrequency, Nerve blocks, Ultrasound guided injection

## Abstract

The aim of this study was to compare the effectiveness of greater occipital nerve (GON) block and pulsed radiofrequency (PRF) treatment in chronic migraine patients. Seventy patients admitted to the Neurology and Algology outpatient clinic between September 2023 and December 2023 and diagnosed with chronic migraine according to The International Classification of Headache Disorders 3rd Edition (ICHD-3) criteria were included in the study. Patients were randomized into 2 groups to receive ultrasound-guided repeated GON block and PRF. Visual Analog Scale (VAS) scores for pain relief and Migraine Disability Assessment (MIDAS) scores for disability were recorded before the procedure and at 1st and 6th months after the procedure. In both groups, 35 patients with greater occipital nerve (GON) block, 32 patients with GON PRF, the pain scores at 1st and 6th months post-procedure were significantly lower compared to before the procedure (*p* < 0.001, *p* < 0.001, respectively). VAS scores were significantly lower in the PRF group than in the GON block group at 6th month (*p* = 0.009). In both groups, post-procedural MIDAS scores at 1st and 6th months were significantly lower compared to before the procedure (*p* < 0.001, *p* < 0.001, respectively). In the GON PRF group, MIDAS scores at 6th month were significantly lower than MIDAS scores at 1st month (*p* < 0.001). MIDAS scores were significantly lower in the PRF group compared to the GON block group at 6th months (*p *< 0.001).Interventional procedures such as GON block and PRF are safe and effective methods in chronic migraine. PRF is a better alternative to GON block in chronic migraine with longer effectiveness.

## 1. Introduction

Migraine is one of the common causes of primary headaches. The incidence of migraine is 18% in men and 43% in women [1]. Chronic migraine is defined as the occurrence of headache for 15 or more days a month for more than 3 months and at least 8 of these pains have migraine-type pain characteristics [2]. Chronic migraine has a prevalence of 1.4% to 5% in the general population [3]. Chronic migraine is a health problem that requires treatment because it affects daily life activities and leads to disability. Peripheral nerve blocks and radiofrequency treatments can be applied in chronic migraine patients who cannot control pain under medical treatment. Greater occipital nerve (GON) block is a common technique in episodic and chronic migraine patients, and its effectiveness has been shown in controlled studies [4, 5, 6, 7].

Pulsed radiofrequency (PRF) is a technique that shows a neuromodulatory effect without neurodestructive properties [8]. Mechanism is based on the transmission of waves from the radiofrequency current provider to tissues through a needle or electrode. In this way, it creates a low-density electric field and causes a decrease in the transmission of pain pathways through myelin-free C-fibers [9, 10]. Although PRF has been studied in various types of chronic pain, its consequences in chronic headaches have not been adequately investigated.

There are a limited number of studies demonstrating the efficacy of PRF treatment. There are few studies covering chronic headaches (migraine, tension type, cluster, occipital neuralgia) [11, 12] and specifically involving migraine patients [13].

In the literature, there is no study comparing the effectiveness of PRF with nerve blocks in isolated migraine patients.

In this randomized controlled design study, we specifically aimed to compare PRF with GON block in terms of pain relief and improvement in disability in chronic migraine patients.

## 2. Materials and methods

The study was designed as a randomized, controlled study comparing the efficacy of PRF and GON blocks. The study was registered to ClinicalTrials (Protocol ID: 2023/03, Clinicaltrials.gov ID: NCT06247592). The design and process of this study are shown with diagram in Fig. 1.

**Fig. 1.** Flow chart diagram. GON: greater occipital nerve; PRF: pulse radiofrequency; VAS: Visual Analog Scale; MIDAS: Migraine Disability Assessment.

### 2.1 Patient selection

Patients between the ages of 18–60 who applied to the Neurology and Algology outpatient clinic between September 2023 and December 2023 and were diagnosed with chronic migraine according to ICHD-3 criteria (Headache occurring on 15 or more days/month for more than 3 months, which, on at least 8 days/month, has the features of migraine headache). Patients who resistant to medical treatment were included in the study.

People with migraine comorbidity (migraine-related silent infarction, prolonged aura, migraine-related seizure, migraine status) were excluded from the study.

People with chronic migraine with known pregnancy, major psychiatric disease, bleeding diathesis, infection in the procedure area, local anesthetic allergy, and patients with cardiac pacemaker were excluded from the study. Patients who underwent surgery from the cranial region within the last 1 year were excluded from the study.

### 2.2 Randomization

70 participants were randomized in a 1:1 ratio with computer-generated randomization tables.

Participants were randomly divided into two groups using the numbered envelope method. Assessment forms were completed by a single blinded observer for the intervention groups prior to the procedure and at 1 and 6 months post-procedure.

### 2.3 Outcome measurements

#### 2.3.1 Primary endpoint

The pre-procedure and post-procedure (for the 1st and 6th months) Visual Analogue Scale (VAS) scores of the patients were evaluated.

#### 2.3.2 Secondary endpoint

Patients’ quality of life and disability were assessed using the Migraine Disability Assessment (MIDAS) before and after the procedure (1 and 6 months).

#### 2.3.3 VAS

It is a scale used to monitor pain severity. A bar or line is given as a scale on which the patient marks the severity of pain as a distance. The patient rates pain on a scale of 0 (none) to 10 (the strongest pain imaginable) [14].

#### 2.3.4 MIDAS

It is a questionnaire applied to measure disability due to headache. Grade I: no disability at all; Grade II: mild disability; Grade III: moderate disability; Grade IV: severe disability [15].

### 2.4 Interventional procedure

All procedures were performed under sterile conditions under ultrasound guidance while the patient was in the prone position. The ultrasound probe was placed in a transverse plane 2–3 cm lateral to the protuberantio ocipitalis externa. After the occipital artery was visualized (Fig. 2), the GON was aimed to be medial to the artery (Fig. 2). After local anesthesia was provided with 1% subcutaneous lidocaine, a 21 Gauge 5 cm long needle tip for the block was directed with an in-plane approach from lateral to medial to place it exactly in the center of the nerve (Fig. 2). After negative aspiration, a block was performed with 3 mL of 2% Prilocaine 1 time per week for 4 weeks.

**Fig. 2.** The images of GON block and PRF procedure with ultrasound-guided in-plane approach. (A) Ultrasonographic image of the location of the occipital artery marked with the red arrow. (B) Ultrasonographic image of the needle placed medially to the occipital artery marked red arrow. (C) Ultrasonographic image of the final needle location for GON block and PRF intervention. N: Needle.

For radiofrequency, the 5 cm long 5 mm active-tip radiofrequency (RF) cannula was placed on the target with ultrasound guidance and then directed with an in-plane approach from lateral to medial to place the tip of the needle exactly in the center of the nerve (Fig. 2). 50 Hz 1V sensory and 2 Hz 2V motor electrical stimulation was performed to reveal compatible paresthesia response in the occipital nerve distribution. Pulse radiofrequency (Neurotherm NT1100/13001-12) was applied at 42 °C for 240 seconds. After the procedure, the patients were taken into observation.

### 2.5 Statistical analysis

The research data were evaluated using the SPSS 21.0 statistical program (IBM, New York, USA). The conformity of continuous variables to normal distribution was investigated using visual (histogram and probability graphs) and analytical methods (Kolmogorov-Smirnov/Shapiro-Wilk tests). Descriptive statistics of the study were summarized using number (n), percentage (%), mean, standard deviation (SD), median, minimum and maximum. Chi-Square Test was used to show whether there was a difference between categorical variables in the study. The Student-*t* Test was used to compare the parametric properties of continuous variables in independent groups, the Mann Whitney U Test was used to compare the non-parametric properties of continuous variables in independent groups, and the Wilcoxon Test or Friedman Test was used to compare the non-parametric properties of continuous variables in dependent groups. Analyses of variances (ANOVA) for repeated measures was used. For statistical significance, a *p*-value lower than 0.05 was set.

## 3. Results

The results of 67 patients (55 females, 12 males) were evaluated. GON block was applied to 35 patients and GON PRF was applied to 32 patients. The demographic characteristics of both groups are given in the table (Table 1).

**Table 1.** Comparison of demographic and clinical characteristics of the groups.

		GON Block	GON PRF	*p*
Gender, n (%)				
	Female	25 (71.4)	30 (93.8)	0.017
	Male	10 (28.6)	2 (6.3)	
Age, Mean ± SD		41.3 ± 12.6	44.8 ± 9.6	0.216*
Medication overuse headache, n (%)				
	(−)	20 (57.1)	11 (34.4)	0.062
	(+)	15 (42.9)	21 (65.6)	
MIDAS 0, n (%)				
	1.00	0	1 (3.1)	0.163
	2.00	6 (17.1)	11 (34.4)	
	3.00	18 (51.4)	10 (31.3)	
	4.00	11 (31.4)	10 (31.3)	
VAS 0, Mean ± SD		7.71 ± 0.79	7.97 ± 1.06	0.399**
Migraine prophylaxis, n (%)				
	(−)	5 (14.3)	7 (21.9)	0.418
	(+)	30 (85.7)	25 (78.1)	
Migraine prophylaxis drugs, n (%)				
	B-Blocker	5 (16.7)	13 (32.5)	0.541
	SNRI	10 (33.3)	10 (25.0)	
	CCB	3 (10.0)	2 (5.0)	
	TCA	10 (33.3)	11 (27.5)	
	Anticonvulsan	2 (6.7)	4 (10.0)	

*Parametric test; **Non parametric test. SNRI: Serotonin-Neuradrenaline reuptake inhibitor; CCB: Calcium channel blocker; TCA: Tricyclic antidepressants; GON: greater occipital nerve; PRF: pulse radiofrequency; SD: standard deviation; MIDAS: Migraine Disability Assessment; VAS: Visual Analog Scale.

In both groups, post-procedural pain scores at 1st month and 6th months were significantly lower compared to before the procedure (sincerely *p* < 0.001, *p* < 0.001) (Table 2). In the GON block group, VAS scores at 1st month were significantly lower than those at 6th month (*p* < 0.001) (Table 2). In both groups, post-procedural MIDAS scores at 1st month and 6th months were significantly lower compared to before the procedure (sincerely *p* < 0.001, *p* < 0.001) (Table 3).

**Table 2.** Comparison of pre-procedure VAS scores with post-procedure scores at 1st month and 6th months.

		VAS 0	VAS 1st month	VAS 6th month	*p*
GON Block					
	Mean	7.7	3.5	4.0	<0.001^1,2,3^
	SD	0.8	1.3	1.4	
	Median	8.0	3.0	4.0	
	Minimum	6.0	2.0	1.0	
	Maximum	9.0	8.0	8.0	
GON PRF					
	Mean	8.0	3.8	3.2	<0.001^1,2,3^
	SD	1.1	1.8	1.2	
	Median	8.0	4.0	3.0	
	Minimum	6.0	1.0	1.0	
	Maximum	10.0	8.0	6.0	

^1^ *There is a statistically significant difference between VAS 0 and VAS 1st month; ^2^There is a statistically significant difference between VAS 0 and VAS 6th month; ^3^There is a statistically significant difference between VAS 1st month and VAS 6th month. VAS: Visual Analog Scale; GON: greater occipital nerve; SD: standard deviation; PRF: pulsed radiofrequency.*

**Table 3.** Comparison of pre-procedure MIDAS scores with post-procedure scores at 1st month and 6th months.

		MIDAS 0	MIDAS 1st month	MIDAS 6th month	*p*
GON Block					
	Mean	3.1	1.7	2.0	<0.001^1,2^
	SD	0.7	0.7	0.3	
	Median	3.0	2.0	2.0	
	Minimum	2.0	1.0	1.0	
	Maximum	4.0	3.0	3.0	
GON PRF					
	Mean	2.9	1.6	1.3	<0.001^1,2,3^
	SD	0.9	0.7	0.5	
	Median	3.0	1.5	1.0	
	Minimum	1.0	1.0	1.0	
	Maximum	4.0	3.0	2.0	

^1^ *There is a statistically significant difference between MIDAS 0 and MIDAS 1th month; ^2^There is a statistically significant difference between MIDAS 0 and MIDAS 6th month; ^3^There is a statistically significant difference between MIDAS 1st month and MIDAS 6th month. MIDAS: Migraine Disability Assessment; GON: greater occipital nerve; SD: standard deviation; PRF: pulsed radiofrequency.*

When comparing both groups, there was no significant difference in VAS scores at 1st month (*p* = 0.2) (Table 4). VAS scores in the PRF group were significantly lower than those in the GON block group at 6th months (*p* = 0.009) (Table 4). In the GON PRF group, MIDAS scores at 6th month were significantly lower than MIDAS scores at 1st month (*p* < 0.001). When comparing both groups, there was no significant difference in MIDAS scores at 1st month. MIDAS scores in the PRF group were significantly lower than GON block group at 6th month, (*p* < 0.001) (Table 4).

**Table 4.** Comparison of VAS and MIDAS scores of both groups before and 1st month and 6th month after the procedure.

	GON Block					GON PRF					*p*
	Mean	SD	Median	Minimum	Maximum	Mean	SD	Median	Minimum	Maximum	
VAS 0	7.7	0.8	8.0	6.0	9.0	8.0	1.1	8.0	6.0	10.0	0.399*
VAS 1	3.5	1.3	3.0	2.0	8.0	3.8	1.8	4.0	1.0	8.0	0.264*
VAS 6	4.0	1.4	4.0	1.0	8.0	3.2	1.2	3.0	1.0	6.0	0.009*
MIDAS 0	3.1	0.7	3.0	2.0	4.0	2.9	0.9	3.0	1.0	4.0	0.273*
MIDAS 1	1.7	0.7	2.0	1.0	3.0	1.6	0.7	1.5	1.0	3.0	0.325*
MIDAS 6	2.0	0.3	2.0	1.0	3.0	1.3	0.5	1.0	1.0	2.0	<0.001*

*Non parametric test. GON: greater occipital nerve; PRF: pulsed radiofrequency; SD: standard deviation; VAS: Visual Analog Scale; MIDAS: Migraine Disability Assessment.

Regarding analgesic consumption at 1st month and 6th month post-procedure, there was no significant difference between the two groups (*p* = 0.83 and *p* = 0.06, respectively) (Table 5).

**Table 5.** Comparison of analgesic withdrawal at 1st and 6th months after the procedure in both groups.

		GON Block	GON PRF	*p*
Analgesic Withdrawal, 1st month				
	(−)	14 (40.0)	12 (37.5)	0.834
	(+)	21 (60.0)	20 (62.5)	
Analgesic Withdrawal, 6th month				
	(−)	22 (62.9)	13 (40.6)	0.069
	(+)	13 (37.1)	19 (59.4)	

*GON: greater occipital nerve; PRF: pulsed radiofrequency.*

## 4. Discussion

Chronic migraine is a health problem that must be treated, which affects the quality of life by causing serious disability. Many medical treatments are insufficient in chronic migraine, which is a persistent pain syndrome. In these cases, GON block, one of the interventional pain treatments, is an effective method applied in chronic migraine patients. Its effectiveness has been shown in many studies. PRF application to the occipital nerve, which is a new treatment option, is an alternative method to GON block in chronic migraine patients. Although there are studies reporting efficacy for PRF in many chronic pain syndromes, there are limited studies on headaches. In our study, we demonstrated that PRF therapy showed similar efficacy to GON blocks at 1st month, but provided better pain relief compared to GON blocks at 6th month. This study is the first randomized controlled trial comparing both techniques in isolated chronic migraine patients. A recent comparative study has shown that non-invasive PRF has similar efficacy with GON blocks in chronic migraine [16]. In this study, a non-invasive PRF technique was investigated, and the results were short-term monitored over a 4-week follow-up period. Similarly, in the early follow-ups (1st month), both techniques showed comparable efficacy in our study. Based on our results, we believe that the effectiveness of local anesthetics in blocks decreases at 6th month follow-up, and PRF may provide better outcomes in long-term pain control through its neuromodulatory effect.

Guner *et al*. [13] showed that PRF administered with ultrasound from the proximal level to the occipital nerve in chronic migraine was effective in terms of pain, quality of life, depression and sleep quality in 3 months follow-up. In our study, we evaluated the quality of life with a scale in addition to pain relief and revealed the importance of evaluating chronic migraine in terms of both pain and disability.

Cohen *et al*. [11] showed that 3 cycles of 120 second PRF treatment applied to a group of occipital neuralgia and migraine patients was superior to steroids at 6 months of follow-up. Preclinical studies are unclear on whether multiple PRF cycles improve treatment outcomes [17, 18]. One clinical study showing a small additional benefit when multiple cycles are administered [19]. Continuation of the activity in 6 months follow-up may be related to 3 cycles of application. In our study, by applying a single cycle PRF, we found that the activity continued on the 3rd month. Cohen *et al*. [11] performed their interventions with anatomical landmark technique without imaging guidance. We applied the interventions by aiming to reach the greater occipital nerve proximally with ultrasound guidance and imaging the occipital artery. Ultrasound-guided interventional procedures offer safer injection since the vascular structures are visualized. In addition, more effective results are obtained since the needle is advanced by seeing the target tissue in ultrasound-guided interventions. Batistaki *et al*. [12] demonstrated the effectiveness of PRF applied using anatomical landmarks in a group of patients with chronic headaches (chronic migraine, cluster headache, occipital neuralgia, tension-type headache) in 6 months follow-up. In the analysis conducted between the groups, no significant difference was shown in terms of effectiveness, and it was concluded that PRF would be an effective method in chronic headaches. Unlike these studies, we compared the effectiveness of two different interventional methods that we used specifically only in chronic migraine patients.

GON block treatment is a very effective interventional method in chronic migraine patients. It significantly affects pain and quality of life in patients. Although local anesthetics provide long pain relief due to their anti-inflammatory activities as well as their block effect, their effectiveness begins to decrease at 6 months. As we confirmed in our study, although they show similar efficacy in the early period, the effectiveness of PRF treatment stands out in long-term follow-up.

PRF exhibits a known neuromodulatory effect due to its non-destructive properties, and the final temperature of the active tip does not exceed 42 °C [20]. It acts through a low-intensity electric field that leads to reduced conduction in pain pathways. Its main effect is on myelin-free C-fibers and does not affect myelinated fibers [21]. Studies have shown that PRF leads to long-term analgesia by significantly modulating synaptic conduction and facilitates the inhibitory effect of serotonergic, noradrenergic, and endogenous opioid pain pathways [22]. PRF is thought to exhibit antinociceptive activity on pain pathways not only through peripheral but also through a central modulation.

Our study showed that PRF is more effective than GON blocks in the treatment of chronic migraine at the 6th month, supporting previous research. In addition to pain relief in chronic migraine, quality of life is also an important evaluation criterion. In our study, we evaluated the quality of life as well as pain scales for the effectiveness of interventional procedures. In both interventional procedures, we found that disability decreased in the early period and quality of life increased similarly.

Our study has some limitations. Small sample size is the first limitation. Secondly, the absence of a blinding method for patients may reduce the reliability of the results due to the placebo effect.

## 5. Conclusions

As a result, interventional procedures are an effective option in chronic migraine when many medical treatments are unresponsive. Interventional procedures such as GON block and PRF in chronic migraine are pleasing in appropriate patients when applied with the right techniques. PRF is an effective and safe option for long-term pain relief in chronic migraine and is a better alternative to GON blocks, whose effectiveness is known. Our study will make significant contributions to the literature by supporting the results of previous studies.
